# Einfluss der Behandlungserwartung auf Pruritus und Hautschmerzen

**DOI:** 10.1007/s00482-021-00600-2

**Published:** 2021-10-27

**Authors:** F. Krefting, S. Hölsken, M. Schedlowski, W. Sondermann

**Affiliations:** 1 Klinik für Dermatologie, Venerologie und Allergologie, Universitätsklinikum Essen, Universität Duisburg-Essen, Hufelandstr. 55, 45122 Essen, Deutschland; 2 Institut für Medizinische Psychologie und Verhaltensimmunbiologie, Universitätsklinikum Essen, Universität Duisburg-Essen, Hufelandstr. 55, 45122 Essen, Deutschland; 3 Department of Clinical Neuroscience, Osher Center for Integrative Medicine, Karolinska Institute, 171 77 Stockholm, Schweden

**Keywords:** Hauterkrankungen, Schmerztherapie, Arzt-Patienten-Beziehung, Placeboeffekt, Noceboeffekt, Skin diseases, Pain management, Physician–patient relationship, Placebo effect, Nocebo effect

## Abstract

**Hintergrund:**

Patientenerwartungen in Bezug auf den Nutzen einer medizinischen Behandlung stellen eine wichtige Determinante für die Placeboantwort dar. Sie können Entwicklung und Verlauf von Erkrankungen sowie Wirksamkeit und Verträglichkeit von Therapien maßgeblich beeinflussen. Die Mechanismen, die diese Placebo- und Noceboeffekte vermitteln, wurden bislang am besten auf dem Gebiet der Placeboanalgesie beschrieben. Aber auch in der Dermatologie findet sich eine zunehmende Evidenz dafür, dass verschiedene Symptome wie Schmerzen an der Haut und Pruritus (Jucken) sowie verschiedene dermatologische Erkrankungen durch die Behandlungserwartungen von Patienten moduliert werden können.

**Ziel der Arbeit:**

Das Ziel dieser Arbeit ist die Darstellung der aktuellen Datenlage in Bezug auf den Einfluss von Erwartungseffekten auf dermatologische Symptome wie Pruritus und Hautschmerzen sowie auf verschiedene dermatologische Erkrankungen. Schließlich soll die Bedeutung dieses Themas für Ärzte, die Patienten mit Hautsymptomen behandeln, vermittelt werden.

**Material und Methoden:**

Es handelt sich um eine narrative Übersichtsarbeit.

**Ergebnisse und Diskussion:**

Eine zunehmende Anzahl von Studien an gesunden Probanden und dermatologischen Patienten zeigt, dass Hautsymptome wie Pruritus und Schmerzen durch die Induktion positiver Erwartungen verringert und durch die Induktion negativer Erwartungen verstärkt werden können. Vorherige Behandlungserfahrungen der Patienten sowie die Qualität und Quantität der Arzt-Patienten-Kommunikation spielen für die Induktion der Behandlungserwartung eine zentrale Rolle.

**Schlussfolgerung:**

Techniken, die darauf abzielen, positive Erwartungseffekte von Patienten mit Hautsymptomen zu maximieren und negative zu minimieren, sollten in die klinische Routine implementiert werden.

## Abstract (en)

**Background:**

Patients’ expectations in terms of the benefit of a treatment are key determinants of placebo responses and can affect the development and course of medical conditions as well as the efficacy and tolerability of active medical treatment. The mechanisms mediating these placebo and nocebo effects have been best described in the field of experimental pain and placebo analgesia. However, also in dermatology experimental and clinical studies demonstrate that different skin symptoms such as itch, skin pain and dermatologic diseases can be modulated by patients’ expectations.

**Objectives:**

The aim of this review is to provide a current overview of the empirical evidence for the effects of patients’ expectations in the field of dermatology with a focus on different skin symptoms such as itch and pain. Finally, the relevance of this topic for physicians who treat patients with dermatologic symptoms is discussed.

**Materials and methods:**

The article is a narrative review.

**Results:**

Steadily growing evidence from experimental and clinical studies in healthy volunteers and dermatologic patients suggests that patients’ positive treatment expectations can reduce skin disease symptoms and enhance treatment efficacy, while negative treatment expectations can induce a nocebo effect associated with increased symptomatology. Patients’ prior treatment experiences as well as the quality and quantity of doctor–patient communication play a central role in shaping treatment expectations.

**Conclusions:**

Techniques aimed at maximizing positive expectation effects in patients should be implemented in daily clinical routine.

Studien aus dem Bereich der Dermatologie dokumentieren, dass Symptome wie Hautschmerzen und Pruritus maßgeblich durch die Erwartungen von Patienten beeinflusst werden können. Vorangegangene Behandlungserfahrungen spielen dabei eine zentrale Rolle. Wenn Patienten die Wirksamkeit eines Medikaments schon mehrfach erlebt haben, wird sich die resultierende positive Erwartung auch günstig auf zukünftige Behandlungen auswirken. Im Gegensatz dazu können negative Vorerfahrungen und Ängste die Wahrscheinlichkeit einer Symptomaggravation im Rahmen von Noceboeffekten erhöhen [4, 5, 9, 15, 22].

## Pruritus und Hautschmerzen

Pruritus ist eine Sinnesempfindung im Bereich der Haut oder Schleimhäute verbunden mit dem Drang zu kratzen. Es handelt sich um ein häufiges fächerübergreifendes Symptom, das ein Leitsymptom vieler Dermatosen ist, wie etwa der atopischen Dermatitis oder Psoriasis. Ähnlich wie chronischer Schmerz wird (chronischer) Pruritus neben somatischen Einflüssen maßgeblich von kognitiven und emotionalen Faktoren beeinflusst und kann die Lebensqualität von Patienten massiv beeinträchtigen [32]. Im Falle der atopischen Dermatitis leiden beispielsweise nahezu 100 % der Patienten unter Pruritus, was zu signifikanten Einschränkungen der Lebensqualität und zu vermehrtem Auftreten von Schlafstörungen, Angstzuständen, Depressionen und Suizidgedanken führt [33]. Oftmals mündet der Pruritus bei diesen Patienten in einen Juck-Kratz-Zyklus: Der Pruritus führt zu Kratzen, was kurzfristig zu einer Symptombesserung führt, das Kratzen verursacht jedoch wieder vermehrte entzündliche Prozesse in der Haut, was dann wiederum vermehrtes Jucken verursacht [26]. Pruritus kann zudem im Rahmen von internistischen, neurologischen oder psychischen Erkrankungen entstehen [32] und ist eine der häufigsten Nebenwirkungen von Schmerzmedikamenten (μ-Opioid-Agonisten; [27]), sodass auch Schmerztherapeuten nicht selten mit dem Symptom Pruritus bei ihren Patienten konfrontiert sind.

Schmerz und Pruritus teilen viele Mechanismen der neuralen Signalverarbeitung

Während Schmerz und Pruritus lange als antagonistische Empfindungen betrachtet wurden, wissen wir heute, dass die beiden Sinnesempfindungen viele Mechanismen der neuralen Signalverarbeitung teilen. So können dieselben Stoffe, abhängig vom Modus der mechanischen Stimulation, sowohl Schmerz als auch Pruritus auslösen, und für beide Empfindungen finden sich ähnliche Muster bei der Sensitivierung zentraler Nervenfasern. Im klinischen Alltag berichten Patienten nicht selten von Mischungen aus Jucken und Schmerz wie „brennendem Jucken“ oder „juckendem Stechen“ [27]. Da Dermatologen in der Regel eher nach Pruritus und Neurologen oder Anästhesisten eher nach Schmerzsymptomen fragen, ist zu erwarten, dass diese klinische Überschneidung zwischen Schmerz und Pruritus nicht immer aufgedeckt wird [27].

Bei Schmerzen an der Haut handelt es sich um Oberflächenschmerz, der von oberflächlich in der Haut gelegenen Nozizeptoren wahrgenommen wird. Jeweils über 40 % der Patienten mit Psoriasis und atopischer Dermatitis leiden unter Hautschmerzen, was einen signifikant negativen Einfluss auf die Lebensqualität hat [16]. Im Vergleich zu gesunden Probanden ist bei Patienten mit atopischer Dermatitis die Schmerzschwelle reduziert, was darauf hindeutet, dass hier eine generalisierte Sensitivierung eine Rolle spielt [28]. Der Erforschung von Hautschmerzen, insbesondere im Rahmen von Dermatosen, wurde jedoch bislang wenig Aufmerksamkeit geschenkt, sodass im Vergleich zum Pruritus deutlich weniger Daten verfügbar sind.

## Behandlungserwartungen

Sowohl klinische als auch experimentelle Untersuchungen belegen, dass die Erwartungen von Patienten bezüglich einer medizinischen Intervention die Wirksamkeit und Verträglichkeit medizinischer Behandlungen erheblich mit beeinflussen und dass diese Behandlungserwartungen maßgeblich für Placeboeffekte verantwortlich sind. Die Behandlungserwartungen werden wesentlich von verbalen Informationen, sozialem Beobachtungslernen und insbesondere von Vorerfahrungen der Patienten beeinflusst. Wenn ein Patient beispielsweise erfahren hat, dass Antihistaminika ihm schon oft gegen die Symptome seiner allergischen Rhinitis oder Urtikaria geholfen haben, und er dann ein Placebo einnimmt, das er für ein Antihistaminikum hält, ist ein positiver Effekt wahrscheinlich. Darüber hinaus nehmen die Arzt-Patienten-Kommunikation sowie kontextuelle Faktoren wie der Arztkittel, die Behandlungsumgebung oder die Applikationsform Einfluss auf die Behandlungserwartung [4, 5, 9, 21]. So fanden sich in der Analyse von Medikamentenstudien bei Patienten mit allergischer Rhinitis stärkere Placeboreaktionen, wenn die Medikation subkutan verabreicht wurde, verglichen mit sublingualer Applikation [21].

Es wird angenommen, dass pharmakologische und psychologische Effekte additiv wirken; das heißt, die Wirkung eines verabreichten Medikaments setzt sich aus der rein pharmakologischen Komponente und den Auswirkungen von Faktoren wie der Behandlungserwartung zusammen. Das zeigt sich in Studien, in denen die Medikamentengabe verdeckt erfolgt, wodurch die psychologische Komponente ausgeschaltet und das Medikament weniger wirksam wird [24].

Noceboeffekte vermutlich für einen Großteil unerwünschter Medikamentenwirkungen verantwortlich

**Figure 1:**
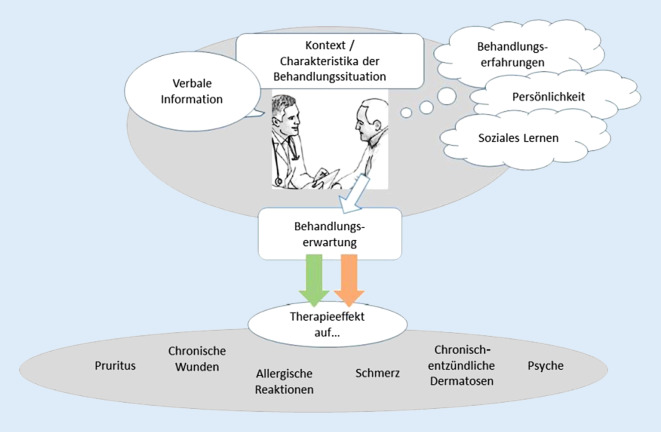

Basierend auf assoziativen Lerneffekten kann eine positive Vorerfahrung mit einem Medikament eine positive Erwartung des Patienten und auf diesem Wege den Placeboeffekt auslösen. Die wiederholte Assoziation eines neutralen Stimulus mit dem unkonditionierten Stimulus führt zur sogenannten konditionierten Reaktion. Dieser Sonderfall der gelernten Placeboantwort wird als „pharmakologische Konditionierung“ bezeichnet [6, 11]. Allerdings können negative Vorerfahrungen, Ängste und Sorgen auch die Wahrscheinlichkeit für unerwünschte Ereignisse im Kontext von Therapien erhöhen. Im klinischen Alltag sind diese sogenannten Noceboeffekte vermutlich für einen Großteil unerwünschter Medikamentenwirkungen verantwortlich ([4, 5, 9, 15, 22]; Abb. 1). Die systematische Nutzung von Erwartungseffekten und die Etablierung von Konditionierungsprotokollen könnten auch in der Dermatologie neue Wege zur Optimierung pharmakologischer Behandlungen eröffnen [25].

## Evidenz für den Einfluss von Erwartungseffekten auf dermatologische Symptome und Erkrankungen

Die Mechanismen, die Placebo- und Noceboeffekte vermitteln, wurden bislang am besten auf dem Gebiet der experimentellen Placeboanalgesie bzw. -hypalgesie beschrieben. Inzwischen wird die Relevanz von Placebo- und Noceboeffekten aber auch in der Dermatologie zunehmend deutlich. Eine Metaanalyse klinischer Studien zu unterschiedlichen Dermatosen ergab beispielsweise eine Reduktion des angegebenen Pruritus von 24 % in den Placebogruppen [30]. Darüber hinaus konnte die Patientenerwartung im dermatologischen Kontext in experimentellen Studien durch assoziative Lernprozesse bzw. Konditionierung, verbale Suggestion oder soziales Lernen manipuliert werden.

### Assoziative Lernprozesse

Evidenz für die Bedeutung assoziativer Lerneffekte kommt beispielsweise aus einer Studie mit Hausstaubmilbenallergikern [10]. Hier wurde im Rahmen eines Konditionierungsparadigmas die Gabe eines Antihistaminikums mit einem speziellen Geschmacksreiz gekoppelt. Die konditionierten Patienten zeigten in der darauffolgenden Testung eine verringerte Basophilenaktivierung im Vergleich zu einer nichtkonditionierten Kontrollgruppe. In einem ähnlichen Paradigma fand sich bei gesunden Probanden in der konditionierten Gruppe ein leicht reduzierter Pruritus als Antwort auf eine Histaminiontophorese [17].

Die „placebounterstützte Dosisreduktion“ basiert ebenfalls auf assoziativen Lernprozessen. In einer solchen Studie mit Psoriasis Patienten konnte die benötigte Medikamentendosis reduziert werden, indem 25–50 % der Medikamentengaben durch ein Placebo ersetzt wurden [1].

### Verbale Informationen

Neben assoziativen Lernprozessen werden Behandlungserwartungen von Patienten durch verbale Informationen induziert. So konnte die Suggestion von gesteigertem oder verringertem Pruritus oder Schmerz in Verbindung mit mechanischer Stimulation diese Empfindung bei gesunden Probanden auslösen oder verringern [29]. Der direkte Vergleich von verbaler Information und Konditionierungseffekten legte nahe, dass vor allem die Kombination beider Methoden effektiv ist, um Placebo- bzw. Noceboantworten auszulösen [2]. Diese Befunde passen zu denen einer Studie zur Konditionierung antihistaminerger Effekte bei Hausstaubmilbenallergikern [31]. Sowohl die konditionierte Gruppe als auch die placebokonditionierte Kontrollgruppe zeigten in dieser Studie eine antihistaminerge Wirkung des verabreichten Placebos.

Die Effekte verbaler Information werden auch bei Patienten deutlich, die unter chronischem Pruritus leiden. Bei Patienten mit atopischer Dermatitis, bei denen ein Prick-Test angewendet wurde, war beispielsweise der empfundene Pruritus bei einer als Allergen präsentierten Kochsalzlösung stärker ausgeprägt als bei offen applizierter Kochsalzlösung [20]. Ähnliche Effekte finden sich in Hinblick auf die Verabreichung von Medikamenten. So konnte eine aktuelle Studie zeigen, dass die offene Gabe eines Antihistaminikums experimentell induzierten Pruritus bei Patienten mit atopischer Dermatitis besser unterdrücken konnte als die verdeckte Gabe desselben Medikaments [24]. Dabei blieb der Effekt sogar bestehen, wenn es sich bei dem per Instruktion als „Antihistaminikum“ verabreichten Medikament de facto lediglich um ein wirkstofffreies Placebo handelte. Darüber hinaus konnten Imaginationstechniken im Rahmen eines multidisziplinären Trainingsprogramms den Pruritus bei Patienten mit atopischer Dermatitis signifikant verringern [8].

Auch aus experimentellen Untersuchungen zu Open-label-Placebos, im Rahmen derer Patienten oder Probanden offen mitgeteilt wird, dass sie ein Placebo erhalten bzw. dass mit verbaler Suggestion gearbeitet wird, ergaben sich Hinweise auf die Wirkungskraft der Behandlungserwartung. Es fand sich in den Studien an gesunden Probanden insbesondere eine Reduktion des erwarteten Pruritus [18, 19].

Auch hinsichtlich der Lebensqualität sind Placebointerventionen ein vielversprechendes Feld

Neben der Verringerung von Pruritus ist auch die Steigerung der Lebensqualität bei dermatologischen Patienten ein wichtiges Behandlungsziel. Unter anderem durch die unangenehmen Hautsymptome, die aufwendige Behandlung und nicht zuletzt durch das soziale Stigma, das mit sichtbaren Hautläsionen verbunden ist, ist die Lebensqualität dermatologischer Patienten häufig stark eingeschränkt. Auch hier sind Placebointerventionen ein vielversprechendes Feld. Bei Patienten mit chronischen Wunden im Bereich der Unterschenkel hatte die Modifikation der Behandlungserwartung zwar keinen Effekt auf die Wundheilung; jedoch zeigten Patienten, die erwarteten, ein hochwirksames Wundgel zu erhalten, eine signifikante Verbesserung der wundbezogenen Lebensqualität [14].

### Soziales Lernen und „ansteckendes Jucken“

Ein weiterer Lernmechanismus neben dem assoziativen Lernen ist das soziale Lernen, das auf Observation und Imitation beruht. Dieser Mechanismus spielt unter anderem eine Rolle bei der Induktion von Pruritus. Audiovisuelles Material, wie krabbelnde Insekten oder Kratzgeräusche, oder allein schon der Anblick eines sich kratzenden Menschen kann Pruritus auslösen – ein Phänomen, das als „ansteckendes Jucken“ („contagious itch“) bekannt ist [23]. Der Effekt tritt sowohl bei gesunden Probanden als auch bei Patienten auf, wobei einige Studien nahelegen, dass er bei Patienten, die unter chronischem Pruritus leiden, größer ist.

### Neurobiologische Mechanismen

Bisher wurden die neurobiologischen Signalwege und Hirnaktivierungsmuster, die für Erwartungseffekte in der Dermatologie eine Rolle spielen, nur in wenigen Studien untersucht. Die existierenden Daten zum Thema Pruritus legen eine Assoziation zwischen der Noceboantwort und der Aktivierung von Hirnarealen nahe, die für die somatosensorische Verarbeitung von Pruritus bzw. den Juck-Kratz-Zyklus relevant sind [13, 20].

Durch Videos induziertes ansteckendes Jucken löste eine verstärkte Aktivierung in Bereichen wie der anterioren Insula, dem prämotorischen und primären somatosensorischen Kortex und dem präfrontalen Kortex aus – Hirnareale, denen zum Teil auch eine Rolle bei der durch Empathie ausgelösten Empfindung des Schmerzes anderer zugeschrieben wird [13]. Ähnlich löste die Gabe von Kochsalzlösung als Nocebo im Prick-Test verglichen mit der Open-label-Bedingung eine vermehrte Aktivierung im dorsolateralen präfrontalen Kortex, dem Nucleus caudatus und dem intraparietalen Sulcus aus [20]. Die klinisch relevantere placeboinduzierte Reduktion von Pruritus wurde hingegen bisher nicht neuropsychologisch analysiert.

### Schlussfolgerungen

Zusammenfassend dokumentieren die experimentellen und klinischen Befunde, dass die Erwartungshaltung von gesunden Probanden und Patienten eine große Rolle bei der Entstehung und Reduktion dermatologischer Symptome spielt. Besonders zum Pruritus gibt es hier bereits eine solide Datenbasis. Ein wichtiges Thema für zukünftige Forschungsarbeiten sind die bisher unterrepräsentierten Hautschmerzen. Analog zu Befunden über die Auswirkung von Patientenerwartungen auf chronischen Schmerz bei anderen Krankheitsbildern sowie auf chronischen Pruritus ist zu erwarten, dass sich auch das Empfinden von Hautschmerz durch verbale Suggestion und andere der oben genannten Techniken beeinflussen lässt. Bisher wurde dies jedoch unseres Wissens noch nicht experimentell an Patienten mit Hautschmerzen untersucht.

## Implikationen für die Praxis

Das zunehmende Wissen über die Wirkkraft der Behandlungserwartung von Patienten mit dermatologischen Symptomen bietet für behandelnde Ärzte die Chance, die klinische Versorgung der Patienten weiter zu verbessern. Dabei scheint es zunächst wichtig, ein Bewusstsein dafür zu schaffen, dass jede Kommunikation und nonverbale Interaktion mit dem Patienten das Potenzial hat, positive oder negative Erwartungen auszulösen, was wiederum den klinischen Verlauf der Erkrankung entsprechend beeinflusst [3]. Eine empathische und authentische Arzt-Patienten-Kommunikation ist zentral für die Generierung einer positiven Behandlungserwartung [5, 12]. Des Weiteren kann es hilfreich sein, Konditionierungsprozesse durch die mehrmalige Verbindung des aktiven Medikaments mit multisensorischen Stimuli, beispielsweise visueller, olfaktorischer oder gustatorischer Art, zu fördern [6, 11]. Der Mechanismus des sozialen Lernens kann genutzt werden, indem Patienten sich mit anderen Patienten austauschen oder Videomaterialien von Patienten sehen, die durch die geplante therapeutische Intervention bereits einen positiven Verlauf erfahren haben [3, 5].

Nebenwirkungen können auch in einen positiven Kontext gesetzt werden

Neben der Förderung positiver Behandlungserwartungen ist die Minimierung von Noceboeffekten essenziell, um einen bestmöglichen Therapieverlauf für den Patienten zu erreichen. Die Nennung möglicher unerwünschter Ereignisse einer Therapie ist obligater Bestandteil einer vertrauensvollen Arzt-Patienten-Kommunikation und gehört zur ärztlichen Aufklärungspflicht. Allerdings birgt eine zu ausgedehnte und detailreiche Darstellung aller potenziell eintretenden Nebenwirkungen das Risiko von Noceboeffekten und somit eines vermehrten Auftretens unerwünschter Wirkungen [5]. Zur Minimierung von Noceboeffekten sollte die deutlich überwiegende Wahrscheinlichkeit ausbleibender Nebenwirkungen in den Fokus der ärztlichen Aufklärung gerückt werden, anstatt sich auf den kleineren Anteil der Patienten zu konzentrieren, die Nebenwirkungen erfahren werden [5]. Zudem ist es auch möglich, Nebenwirkungen in einen positiven Kontext zu setzen [12]. So kann das Auftreten einer Nebenwirkung beispielsweise als Zeichen gewertet werden, dass das Medikament besonders gut wirkt und somit auch eine gute Chance für die Linderung der Krankheitssymptome besteht. Eine weitere potenzielle Quelle für Noceboeffekte bergen Medikamentenumstellungen von kostenintensiven Originalpräparaten zu preisgünstigeren Biosimilars, da günstigere Medikamente mit niedrigeren Patientenerwartungen assoziiert sind [7]. Ein vertrauensvoller Arzt-Patienten-Dialog über wissenschaftlich belegte vergleichbare Therapiewirksamkeit und Nebenwirkungen ist zentral, um Noceboeffekten entgegenzuwirken.

## Fazit für die Praxis

- Patientenerwartungen in Bezug auf den Nutzen einer Behandlung stellen einen wichtigen Einflussfaktor der Placeboantwort dar.
- Für die Induktion einer positiven Behandlungserwartung spielen vorherige Behandlungserfahrungen, die ärztliche Kommunikation und eine vertrauensvolle Arzt-Patienten-Beziehung eine Schlüsselrolle.
- Die empirischen Daten deuten darauf hin, dass Erwartungseffekte einen signifikanten Einfluss auf dermatologische Symptome wie Pruritus haben.
- Weitere wissenschaftliche Studien zu Erwartungseffekten im dermatologischen Bereich, etwa bei Patienten mit chronischem Pruritus oder Schmerzen an der Haut, könnten weitere wichtige Erkenntnisse für die klinische Praxis liefern.
- Techniken, die darauf abzielen, positive Erwartungseffekte gezielt zu maximieren und negative zu minimieren, sollten bereits jetzt in die tägliche klinische Routine implementiert werden.

## References

[1] Ader R, Mercurio MG, Walton J (2010). Conditioned pharmacotherapeutic effects: a preliminary study. Psychosom Med.

[2] Bartels DJ, van Laarhoven AI, Haverkamp EA (2014). Role of conditioning and verbal suggestion in placebo and nocebo effects on itch. PLoS One.

[3] Benedetti F (2013). Placebo and the new physiology of the doctor-patient relationship. Physiol Rev.

[4] Benedetti F (2014). Placebo effects: from the neurobiological paradigm to translational implications. Neuron.

[5] Colloca L, Barsky AJ (2020). Placebo and nocebo effects. N Engl J Med.

[6] Doering BK, Rief W (2012). Utilizing placebo mechanisms for dose reduction in pharmacotherapy. Trends Pharmacol Sci.

[7] Espay AJ, Norris MM, Eliassen JC (2015). Placebo effect of medication cost in Parkinson disease: a randomized double-blind study. Neurology.

[8] Evers AW, Duller P, de Jong EM (2009). Effectiveness of a multidisciplinary itch-coping training programme in adults with atopic dermatitis. Acta Derm Venereol.

[9] Evers AWM, Colloca L, Blease C (2018). Implications of placebo and nocebo effects for clinical practice: expert consensus. Psychother Psychosom.

[10] Goebel MU, Meykadeh N, Kou W (2008). Behavioral conditioning of antihistamine effects in patients with allergic rhinitis. Psychother Psychosom.

[11] Hadamitzky M, Lückemann L, Pacheco-López G (2020). Pavlovian conditioning of immunological and neuroendocrine functions. Physiol Rev.

[12] Hansen E, Zech N (2019). Nocebo effects and negative suggestions in daily clinical practice—forms, impact and approaches to avoid them. Front Pharmacol.

[13] Holle H, Warne K, Seth AK (2012). Neural basis of contagious itch and why some people are more prone to it. Proc Natl Acad Sci USA.

[14] Jockenhofer F, Knust C, Benson S (2020). Influence of placebo effects on quality of life and wound healing in patients with chronic venous leg ulcers. J Dtsch Dermatol Ges.

[15] Kaptchuk TJ, Miller FG (2015). Placebo effects in medicine. N Engl J Med.

[16] Ljosaa TM, Mork C, Stubhaug A (2012). Skin pain and skin discomfort is associated with quality of life in patients with psoriasis. J Eur Acad Dermatol Venereol.

[17] Meeuwis SH, van Middendorp H, Pacheco-Lopez G (2019). Antipruritic placebo effects by conditioning H1-antihistamine. Psychosom Med.

[18] Meeuwis SH, van Middendorp H, van Laarhoven AIM (2019). Effects of open- and closed-label nocebo and placebo suggestions on itch and itch expectations. Front Psychiatry.

[19] Meeuwis SH, van Middendorp H, Veldhuijzen DS (2018). Placebo effects of open-label verbal suggestions on itch. Acta Derm Venereol.

[20] Napadow V, Li A, Loggia ML (2015). The imagined itch: brain circuitry supporting nocebo-induced itch in atopic dermatitis patients. Allergy.

[21] Narkus A, Lehnigk U, Haefner D (2013). The placebo effect in allergen-specific immunotherapy trials. Clin Transl Allergy.

[22] Schedlowski M, Enck P, Rief W (2015). Neuro-bio-behavioral mechanisms of placebo and nocebo responses: implications for clinical trials and clinical practice. Pharmacol Rev.

[23] Schut C, Grossman S, Gieler U (2015). Contagious itch: what we know and what we would like to know. Front Hum Neurosci.

[24] Solle A, Worm M, Benedetti F (2021). Targeted use of placebo effects decreases experimental itch in atopic dermatitis patients: a randomized controlled trial. Int J Clin Pharmacol Ther.

[25] Sölle A, Worm M, Klinger R (2016). Placebo- und Nocebo-Reaktionen bei Juckreiz – klinisch relevante Reaktionen?. Allergologie.

[26] Ständer S (2019). How acute stress impacts the itch-scratch cycle in atopic dermatitis: a clinical lesson. Br J Dermatol.

[27] Stander S, Schmelz M (2020). Neuropathic pruritus. Schmerz.

[28] van Laarhoven AI, Kraaimaat FW, Wilder-Smith OH (2007). Generalized and symptom-specific sensitization of chronic itch and pain. J Eur Acad Dermatol Venereol.

[29] van Laarhoven AI, Vogelaar ML, Wilder-Smith OH (2011). Induction of nocebo and placebo effects on itch and pain by verbal suggestions. Pain.

[30] van Laarhoven AIM, van der Sman-Mauriks IM, Donders ART (2015). Placebo effects on itch: a meta-analysis of clinical trials of patients with dermatological conditions. J Invest Dermatol.

[31] Vits S, Cesko E, Benson S (2013). Cognitive factors mediate placebo responses in patients with house dust mite allergy. PLoS One.

[32] Weisshaar E, Szepietowski JC, Dalgard FJ (2019). European S2k guideline on chronic pruritus. Acta Derm Venereol.

[33] Zeidler C, Pereira MP, Huet F (2019). Pruritus in autoimmune and inflammatory dermatoses. Front Immunol.

